# Lumen and mucosa-associated *Lactobacillus rhamnosus* from the intestinal tract of organ donors

**DOI:** 10.1017/gmb.2020.4

**Published:** 2020-11-10

**Authors:** Alan J. Marsh, Al-Mounawara A. Yaya, Sandy Ng, Kshipra Chandrashekhar, Jeff Roach, Scott T. Magness, M. Andrea Azcarate-Peril

**Affiliations:** a Department of Medicine, Division of Gastroenterology and Hepatology, School of Medicine, University of North Carolina, Chapel Hill, NC, USA; b UNC Microbiome Core, Center for Gastrointestinal Biology and Disease, School of Medicine, University of North Carolina, Chapel Hill, NC, USA; c UNC Information Technology Services and Research Computing, University of North Carolina, Chapel Hill, NC, USA; d UNC/NC State University Joint Departments of Biomedical Engineering and UNC Departments of Medicine, Cell Biology & Physiology, University of North Carolina, Chapel Hill, NC, USA

**Keywords:** *Lactobacillus rhamnosus*, Organ Donor, Lumen, Mucosa, Microbiome

## Abstract

Knowledge of the intra-individual spatial and regional distribution of intestinal microbial populations is essential to understand gut host–microbial interactions. In this study, we performed a compositional analysis of luminal and mucosal samples from the small and large intestine of four organ donors by 16S rRNA amplicon sequencing and high-throughput quantitative polymerase chain reaction. Since the human microbiota is subject to selection pressure at lower taxonomic levels, we isolated over 400 bacterial strains and investigated strain-level variation of 11 *Lactobacillus rhamnosus* from different intestinal regions. Results substantiate reported inter-individual variability as well as intra-individual differences along the gastrointestinal tract. Although the luminal and mucosal-associated communities were similar within individuals, relative abundance reflected the donors’ demographic and potential pathologies. The total bacterial load of all donors increased from small intestine to colon, while *Bifidobacterium* was in greater abundance in the small intestine. Comparative genomic analysis of *L. rhamnosus* showed the strains segregated into two distinct clusters and identified no features specific to location. Analysis revealed genetic differences for exopolysaccharide production, carbohydrate utilization, pilus formation and vitamin K biosynthesis between clusters. This study contributes to the understanding of niche-specific microbial communities, encouraging subsequent studies to better understand microbial signatures at lower taxonomic levels.

## Introduction

Host-associated microorganisms play significant roles in systemic disease prevention and maintenance of overall good gut health. These roles include priming our immune system, influencing host metabolism, and providing important metabolites to the host (Marsh & Azcarate-Peril, 2020). The composition and functionality of the gut microbiota is determined by intrinsic (host genetics, age and general health status) as well as extrinsic factors (diet, lifestyle, antibiotic use, mode of birth and breast-feeding).

Sampling of the human gastrointestinal (GI) tract presents logistical and practical challenges (Human Microbiome Project, 2012), hence limited studies have analysed the microbial populations associated with specific regions of the intestine. Such studies have typically been carried out using biopsies (Stearns et al., 2011; Zmora et al., 2018) or inferred from animal models, which may not be directly comparable to humans (Yasuda et al., 2015). More often, fecal material is used as a proxy for characterizing the gut microbiota; however, it is known that microbial communities vary within the intestinal tract with respect to abundance and diversity. The microbial load is greatest in the large intestine, where bacteria are housed at approximately 10^11^ cells/g (Sender et al., 2016). Here, the lumen is strictly anaerobic and the slow transit time allows for undigested dietary polysaccharides to be fermented. In comparison, the small intestine has higher oxygen concentrations, a pH gradient, a single mucosal layer, a more rapid transit time, and a high concentration of bile salts. Furthermore, as the small intestine is the primary site for nutrient absorption and assimilation, bacteria residing here must compete with the host for simple dietary nutrients (Zmora et al., 2018). These attributes ensure lower colonization rates compared to the large intestine.

Reports of the small intestinal microbiome from biopsy samples showed relatively high levels of facultative anaerobic Proteobacteria and representatives of the oral microbiota (Dieterich et al., 2018). In contrast, the colon is dominated by *Lachnospiraceae* (Firmicutes) and Bacteroidetes (Dieterich et al., 2018). Here, the GI mucosa represents an important interface between the dual mucus layer lining the epithelium and the lumen. The second mucus layer is loose and unattached, acting as a habitat for colonic bacteria which can harvest digestible monosaccharides from host-produced glycans, which decorate the mucin protein core (Johansson et al., 2011). It has been shown that the microbiota from mucosal samples can be distinct from that of feces (Stearns et al., 2011; Zmora et al., 2018) and play an important role in innate immunity.

Currently, there is no definition for what constitutes a healthy gut microbiome, in part due to the significant inter-individual variability in microbial composition (Lloyd-Price et al., 2016). Although intestinal diversity is similar between individuals at higher taxonomic ranks (Stearns et al., 2011), the microenvironments contained within the intestine promote a strong selection pressure favouring radial variation at the species and strain levels (Ley et al., 2006). Furthermore, horizontal gene transfer (HGT) is more common in the gut compared to other environments, with approximately 40 per cent of lateral gene transfer events occurring *in situ* (Jeong et al., 2019). This can enhance the potential for rapid acquisition of genes and adoption of new traits (Lerner et al., 2017). The ability for rapid adaptation is also relevant for beneficial gut species. *L. rhamnosus,* an authochthonous member of the gut community, known for its beneficial attributes, contains a core genome of >2,000 genes, but more than 2,500 variable genes (the extended genome) which can impact the biological attributes (eg. carbohydrate metabolism and bacteriocin production) of a given strain, especially as this species has shown a tendency for high levels of HGT (Ceapa et al., 2016).

In this study, we first aimed to characterize intra-individual differences along the GI tract by profiling the bacterial communities of lumen and mucosa from six intestinal regions of four individual organ donors. Our second aim was to determine whether bacterial isolates of the same species had genetic and phenotypic adaptations specific to their niche. To facilitate this analysis, we isolated over 400 strains from one donor and selected 11 *L. rhamnosus* strains from defined intestinal niches. Comparative genomics and phenotypic characterization were used to determine genetic differences beyond the species-level.

## Materials and methods

### Processing of intestinal tissues for microbiome analysis and isolation of bacterial strains

Intestinal samples were transported to the laboratory, on ice, immediately (within 1 h) after organs were retrieved for donation. Samples were dissected into six segments (duodenum, jejunum, ileum, ascending, transverse and descending colon). Upon receipt, the intestinal sections were placed in oxygen pre-reduced Rich media broth plus 15 per cent glycerol and stored at −80°C until the time of processing. Rich media was composed of: glucose (15 g/L), yeast extract (10 g/L), proteose peptone (5 g/L), beef extract (2.5 g/L), 1.0 ml of MgSO_4_ solution (50 mg/ml), NH_4_H_2_PO_4_ (0.50 g/L) and 10 ml hemin (0.5 mg/100 ml). All samples and bacterial isolates were maintained in an anaerobic chamber (Coy Laboratory Products Inc., MI) with a N_2_:H_2_:CO_2_ gas mix ratio of 85:10:5 per cent at 37°C. All media, glycerol and buffers were pre-reduced in the anaerobic chamber for a minimum of 6 h. Samples were serially diluted in sterile phosphate buffered saline (PBS) and plated on Lactobacilli deMan, Rogosa and Sharpe (MRS) agar (Hardy Diagnostics, Santa Maria, CA), Thioglycolate agar (Becton Dickinson, Sparks, MD), and Rich media. Following a 48-h incubation period, triplicates of colonies with distinct colony morphology were selected from each plate. Polymerase chain reaction (PCR) and Sanger sequencing of the full 16S rRNA gene were carried out on single pure colonies using the Universal 16S rRNA primers 16S_27F/16S_1512R (Weisburg et al., 1991).

### 16S rRNA amplicon sequencing

Barcoding and library preparation were carried out as described (Azcarate-Peril et al., 2018; Carlson et al., 2018; Jones et al., 2018). Total DNA was amplified using universal primers targeting the V4 region of the bacterial 16S rRNA gene (Gregory Caporaso et al., 2011). Master mixes contained 12.5 ng of total DNA, 0.5 μM of each primer, and 2× KAPA HiFi HotStart ReadyMix (KAPA Biosystems, Wilmington, MA). Each sample was amplified using a limited cycle PCR program, adding Illumina sequencing adapters and dual‐index barcodes (index 1(i7) and index 2(i5)) (Illumina, San Diego, CA) to the amplicon target. The final libraries were purified using the AMPure XP reagent (Beckman Coulter, Brea, CA, USA), quantified and normalized prior to pooling. The DNA library pool was then denatured with NaOH, diluted with hybridization buffer, and heat-denatured before loading on the MiSeq instrument. Automated cluster generation and paired-end sequencing with dual reads were performed according to the manufacturer’s instructions.

### Bioinformatics analysis

Paired-end fastqs were joined using the Quantitative Insights Into Microbial Ecology (QIIME) software 1.8.0 (Caporaso et al., 2010) invocation of fastq-join. Further bioinformatics analysis of bacterial 16S rRNA amplicon sequencing data were conducted using QIIME. Operational taxonomic units (OTU) picking, detection of chimeric sequences and alpha and beta analyses were performed on the data set using QIIME as described previously (Allali et al., 2017). Summary reports of taxonomic assignment by sample and all categories were produced using QIIME summarize_taxa_through_plots.py and summarize_otu_by_cat.py. Alpha diversity indices (Phylogenetic Diversity, Shannon and species richness) and beta diversity estimations were carried out using QIIME at a rarefaction depth of 5,000 sequences per sample.

### qPCR 24.192 dynamic array

The Access Array AA 24.192 (Fluidigm Corporation, San Francisco, CA) was used to determine total bacterial abundance as well as the genera *Bifidobacterium* and *Lactobacillus* in the intestinal samples (Azcarate-Peril et al., 2017). Primers are listed in Supplemental Table S1. The average of the Ct values from two sets of primers amplifying the 16S rRNA gene was used to determine total bacterial load. Respective Ct values for the 16S rRNA gene were subtracted from those of *Bifidobacterium* and *Lactobacillus* to normalize the data. Statistics were performed with the two-sample *t*-test using Graphpad Prism 8.0 (GraphPad Software, Inc, La Jolla, CA).

### Characterization of L. rhamnosus

To ensure purity, isolates were Gram stained (BBL^TM^ Gram Staining Kit, Becton Dickinson & Company, MD), followed by sequencing of the full 16S rRNA gene. A total of 100 μl of cultures were harvested by centrifugation and washed twice in API®50CHL assay medium (BioMérieux, Marcy-Star, France). Cells were re-suspended in 10 ml of API®50CHL assay medium and, as per manufacturer’s instructions, transferred to API®50CH strips and incubated at 37°C for 48 h, after which carbohydrate fermentation was recorded. Susceptibility to ampicillin (10 μg), penicillin (10 μg), erythromycin (15 μg), tetracycline (30 μg), gentamycin (10 μg) and kanamycin (30 μg) was assayed using the disc diffusion method with BBL^TM^ Sensi-Disc^TM^ (Becton Dickinson & Company, Sparks, MD) according to the manufacturers’ instructions.

*Growth curves and phage induction.* *L. rhamnosus* strains were cultured anaerobically in MRS broth for 18 h. One milliliter of cells were harvested and washed twice in PBS. Cells were diluted 1:100 in MRS without dextrose and supplemented with either 1 per cent glucose or 1 per cent lactose in 200 μl volumes. Readings were taken every 15 min for 24 h using a BioTeck Epoch2 microplate spectrophotometer (Vermont), located inside an anaerobic chamber. For phage induction, cultures were grown in MRS until an OD_600_ between 0.1 and 0.2 was reached, at which point 0.1, 0.3 and 2 μg of mitomycin were added to the cultures. As previously observed (Durmaz et al., 2008), optimal induction occurred with 0.1 μg.

*Scanning electron microscopy (SEM).* Bacterial cells in suspension were centrifuged at 1,500*g*, and the supernatant was removed. Bacterial cell pellets were resuspended in 2 per cent paraformaldehyde/2.5 per cent glutaraldehyde in 0.15 M sodium phosphate buffer, pH 7.4, for 1 h at room temperature and stored at 4°C. The fixed cell suspension was deposited onto 12 mm round poly-d-lysine coated coverslips and following preparation were mounted on 13 mm aluminum stubs and sputter coated with 5 nm of a gold-palladium alloy (60 Au:40 Pd, Cressington Sputter Coater 208HR, model 8000-220, Ted Pella, Redding, CA). Imaging was done with Zeiss Supra 25 FESEM (Carl Zeiss SMT Inc., Peabody, MA).

*Strain DNA isolation and whole genome sequencing.* DNA was isolated using a modified Qiagen DNeasy Blood & Tissue kit (Qiagen, Valencia, CA). First, prior to the addition of proteinase K, 50 μl of 60 mg/ml lysozyme (Thermo Scientific, IL) was added to each tube and incubated at 37°C for 1 h. Prior to the addition of ethanol, samples were bead beaten for 10 min in a Qiagen TissueLyser II at 30 Hz. DNA was eluted in nuclease-free H_2_O and quantified using a Nanodrop (Thermo Scientific). Paired-end sequencing was performed using Thermofisher Ion GeneStudio™ S5.

*Genome analysis.* Genomes were assembled using SPAdes (Bankevich et al., 2012) and the quality of genomes was assessed using Check-M (Parks et al., 2015). Prokka (Seemann, 2014) was used for genome annotation and Rapid Annotation using Subsystem Technology (RAST) was used to annotate genes to a subsystem category (Ramy et al., 2008). Single nucleotide polymorphism (SNP) analysis and phylogenetic trees were constructed using the Harvest suite of core-genome alignment and visualization tools (Treangen et al., 2014). EzBioCloud was used to determine strain average nucleotide identity (ANI) (Yoon et al., 2017). Whole genome alignment and visual comparison of strains were performed using BLAST Ring Image Generator (BRIG) software (Alikhan et al., 2011). Phaster identified intact and incomplete phage within the genomes (Arndt et al., 2016) and the Comprehensive Antibiotic Resistance Database (CARD) was used to screen for antimicrobial resistance genes (Jia et al., 2017). PlasmidFinder 2.1 was used to search for plasmids (Carattoli et al., 2014). Novel regions between the strains were identified using the online tool Panseq (Laing et al., 2010). Presence or absence of novel genes was plotted as heatmaps using OriginLab software (Origin Lab, Northampton, MA). Novel regions between the different genomes were analysed using Geneious software (Kearse et al., 2012), and cross-alignments were performed with Mauve (Darling et al., 2004). Genomes were submitted to GenBank (National Centre for Biotechnology Information) under the project code PRJNA645702. Accession numbers for individual genomes are listed in Supplemental Table S2.

## Results

Intestinal segments from four organ donors were procured from Carolina Donor Services, an organization that supports and provides organs and tissues for transplantation, between 2017 and 2018. Organ donor 1 was a 10-year old female who suffered a cardiovascular event, donor 2 was a 23-year old male who suffered a fatal gunshot wound and organ donors 3 and 4 were respectively a 65-year old female and a 12-year old male who suffered cerebrovascular events.

Lumen and mucosa from sections of the duodenum, jejunum, ileum, ascending, transverse and descending colons were analysed by 16S rRNA amplicon sequencing and high-throughput quantitative PCR (qPCR). Of the 46 samples sequenced, 16 did not yield sufficient sequencing reads and were not included in downstream analyses (Supplemental Table S3). Of the 16 samples, one was from the large intestine; the remaining were all samples from the small intestine. For the remaining 30 samples, a total of 2,022,736 reads were obtained, equivalent to an average of 67,425 reads per sample. Ninety-nine per cent of the sequencing reads were assigned to a taxonomic group while the residual reads were unassigned.

### Inter-individual differences in gut microbial diversity and composition

**Figure 1:**
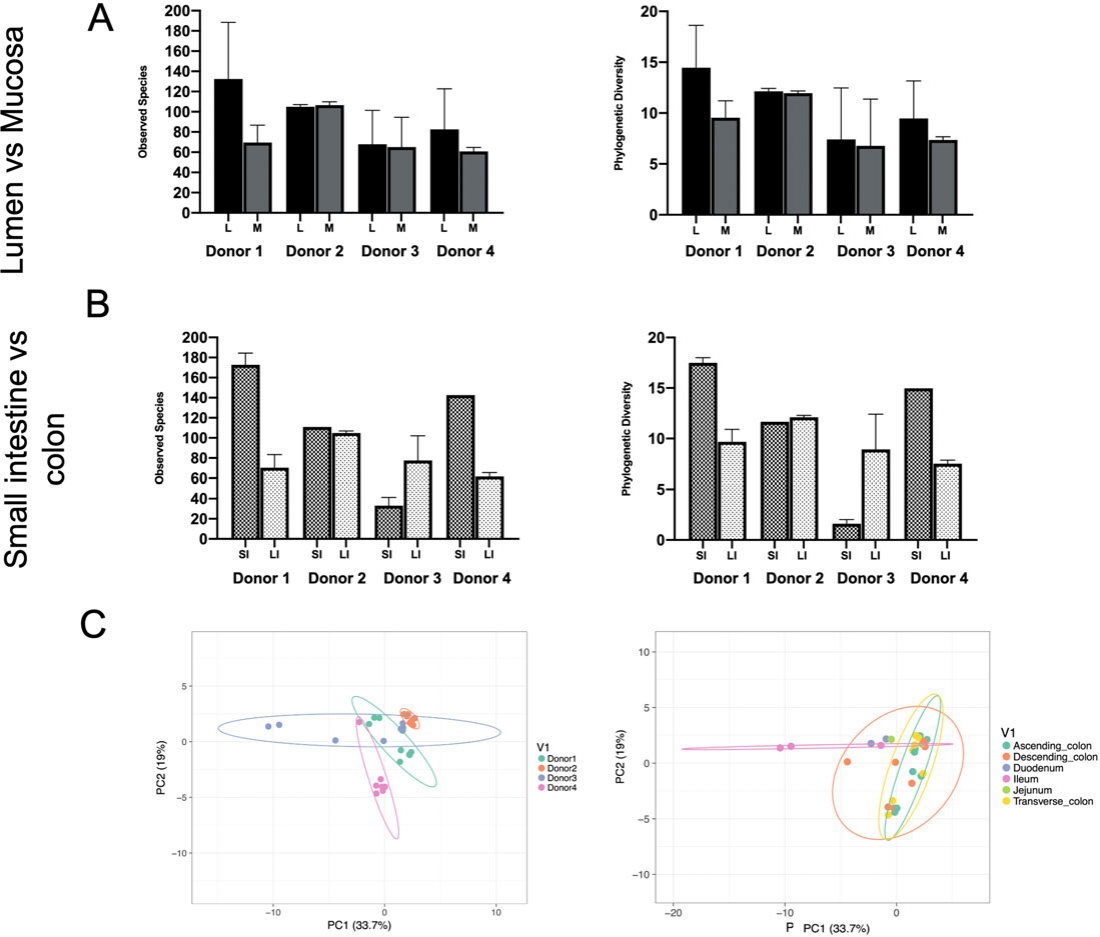
Observed species and phylogenetic diversity comparing donors’ diversity in (A) lumen versus mucosa and (B) small intestine versus large intestine. (C) Principal component analysis (PCA) of samples colored by individual (left) or intestinal segment (right). Unit variance scaling was applied to rows; SVD (singular value decomposition) with imputation is used to calculate principal components. *X* and *Y* axes show principal components 1 and 2 that explain 33.7 and 19 per cent of the total variance, respectively. Prediction ellipses are such that with probability 0.95, a new observation from the same group will fall inside the ellipse. *N* = 30 data points.

Phylogenetic diversity and species richness varied between samples and donors, with the youngest organ donors (1 and 4) presenting with a higher diversity in the small intestine compared to older donors (2 and 3). Additionally, as expected samples clustered by donor rather than by intestinal segments (Figure 1C).

Overall, Firmicutes (represented in greatest abundance by the families *Lactobacillaceae*, *Streptococcaceae*, *Lachnospiraceae* and *Ruminococcaceae*) were more abundant than Bacteroidetes. The most abundant phylum across intestinal segments from donor 1 was Actinobacteria (34.8 per cent), donors 2 and 4 were dominated by Firmicutes (60.8 and 61.4 per cent, respectively), while donor 3 had an over-representation of Proteobacteria (60.1 per cent) (Supplemental Figure S1). Proteobacteria were present in lower abundance (0.9–31.8 per cent) in all samples except in donor 3 where it was represented at >95 per cent in the ileum alone. The most prevalent OTUs were an uncharacterized genus of the *Enterobacteriaceae* family (22.7 ± 21.4 per cent), *Bacteroides* (13.7 ± 10.3 per cent), *Blautia* (9.3 ± 7.7 per cent), *Bifidobacterium* (6.7 ± 11.5 per cent), *Ruminococcus* (3.3 ± 1.4 per cent) and an uncharacterized *Lachnospiraceae* genus (7 ± 3.6 per cent).

**Figure 2:**
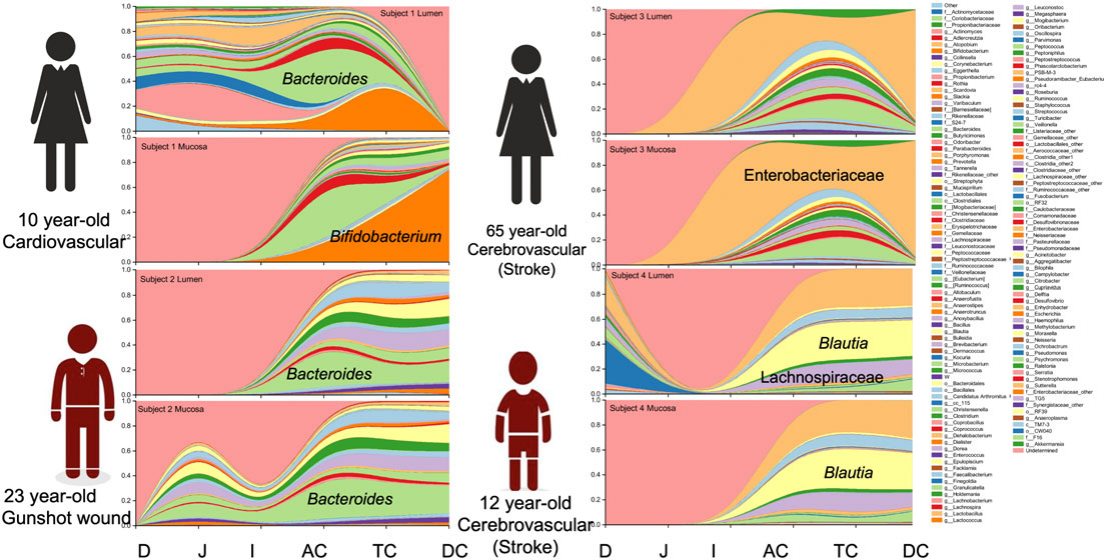
Genus distribution by donor per intestinal section and region.

Notable differences were observed between donors at the genus level. The predominant taxa were *Bifidobacterium* (23.2 per cent) in donor 1, *Bacteroides* (24.0 per cent) in donor 2, an uncharacterized genus of the *Enterobacteriaceae* family (59.9 per cent) in donor 3 and *Blautia* (25.8 per cent) and an uncharacterized *Enterobacteriaceae* genus (24.9 per cent) in donor 4 (Figure 2 and Supplemental Table S4). In addition to the dominant genus in each subject, taxa configuration and the presence of species considered beneficial varied extensively by individual and intestinal region (Supplemental Tables S5 and S6).

### Comparison of mucosal and luminal bacterial populations

In general, mucosal samples of the small intestine yielded low numbers of reads (<1,000 reads/sample). The abundance of Firmicutes were greater in the lumen compared to the mucosa (lumen = 46.5 ± 38.6 per cent vs. mucosa = 41.2 ± 31.3 per cent) across all donors and intestinal regions, while Proteobacteria (lumen = 28.0 ± 42.8 per cent vs. mucosa = 41.2 ± 39.3 per cent), Bacteroidetes (lumen = 17.1 ± 11.1 per cent vs. mucosa = 20.0 ± 14.4 per cent) and Actinobacteria (lumen = 5.4 ± 2.6 per cent vs. mucosa = 12.3 ± 13.2 per cent) had higher average relative abundances in the mucosa. Firmicutes were over-represented in all intestinal sections except in the ileum where Proteobacteria were overrepresented at 67.1 ± 50.0 per cent. This observation is askew due to donor 3, who had an abnormally high representation of Proteobacteria. At the genus level, overall, the predominant groups were an uncharacterized *Enterobacteriaceae* genus (lumen = 20.7 ± 24.4 per cent vs. mucosa = 25 ± 29.1 per cent), *Blautia* (lumen = 8.4 ± 9.5 per cent vs. mucosa =10.4 ± 9.9 per cent), an uncharacterized genus of the family *Lachnospiraceae* (lumen = 6.8 ± 4.1 per cent vs. mucosa = 7.2 ± 4.7 per cent), *Bacteroides* (lumen = 12.3 ± 7.2 per cent vs. mucosa = 15.3 ± 9.2 per cent) and *Bifidobacterium* (lumen = 4.1 ± 6.9 per cent vs. mucosa = 9.5 ± 15.1 per cent).

At the individual level, the most represented phyla in donor 1 were Firmicutes in the lumen and Actinobacteria in the mucosa. In donors 2 and 4, there was increased Firmicutes in the lumen and mucosa compared to the other samples, while donor 3 had a predominance of Proteobacteria. In the lumen, the dominant genus in donors 1 and 2 was *Bacteroides* (16.3 and 22.0 per cent, respectively), whereas donors 3 and 4 exhibited an uncharacterized *Enterobacteriaceae* genus (55.4 per cent) and *Blautia* (22.6 per cent). In the mucosa, the dominant groups were *Bifidobacterium* (42.1 per cent, donor 1), *Bacteroides* (25.4 per cent, donor 2), an uncharacterized genus of *Enterobacteriaceae* (64.4 per cent, donor 3) and *Blautia* (30.2 per cent, donor 4).

### Bacterial load along the GI tract

**Figure 3:**
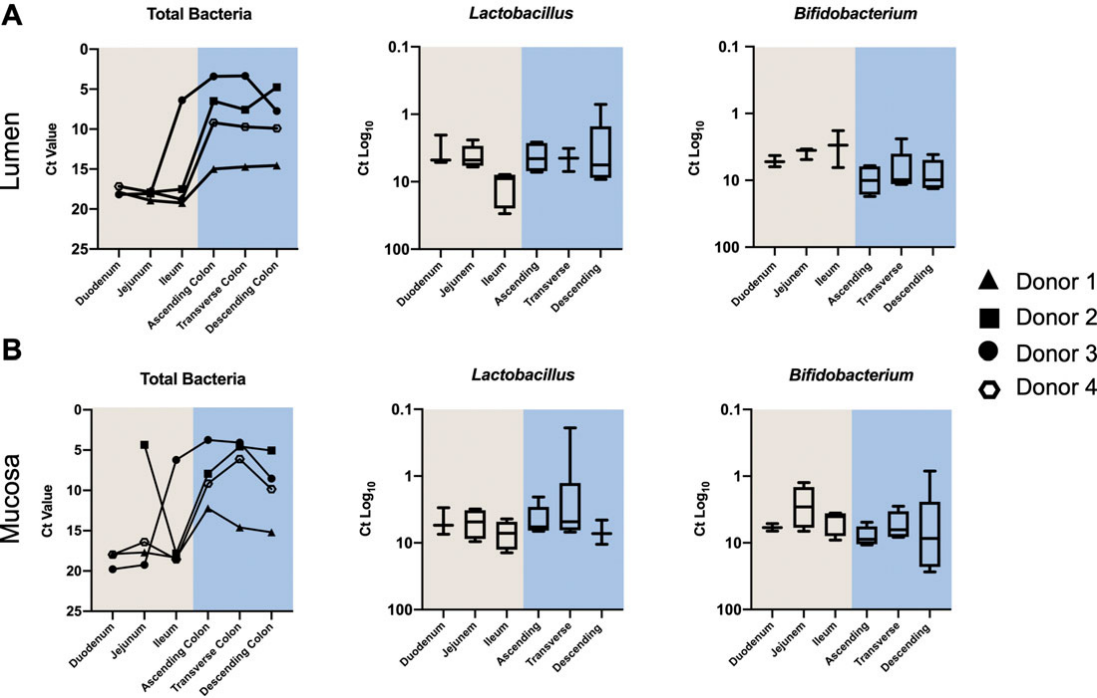
Quantitative polymerase chain reaction (qPCR) data showing relative proportions of total bacteria, *Lactobacillus* and *Bifidobacterium* across the different intestinal segements in (A) the lumen and (B) the mucosa.

A number of intestinal samples did not provide enough sequencing reads to determine their community profile (Supplemental Table S3). Consequently, to obtain abundance information for all samples regarding total bacterial load and the beneficial taxa *Bifidobacterium* and *Lactobacillus,* we conducted high throughput qPCR (Figure 3). Overall, the bacterial load associated with the intestinal mucosa was similar to the lumen. All individuals, in both lumen and mucosa, had higher bacterial loads in the colon (*p* = 0.0001). Patterns were consistent across mucosal and luminal samples for each donor, with donor 1 exhibiting the lowest overall abundance, and donors 2 and 3 the highest. For mucosal samples, all but one donor (donor 2) showed the greatest abundance in the ascending/transverse colon with a decline in the descending colon (*p* = 0.14). A similar trend was observed in the lumen samples for donors 3 and 4, with a marginal increase in the descending colon of donor 1.

*Lactobacillus* qPCR data showed similar Ct values across lumen (6.40 ± 5.64) and mucosa (5.94 ± 2.93) in all donors. However, ileal lumen samples had a lower abundance of *Lactobacillus*. The overall mean Ct value of *Bifidobacterium* was lower in the mucosa (6.75 ± 5.16) than in the lumen (7.04 ± 4.47), indicating a higher proportion of *Bifidobacterium* in the mucosa compared to the lumen. Conversely, the average Ct values for *Bifidobacterium* in the colon (9.03 ± 5.38) were higher than that of the small intestine (4.44 ± 1.95) indicating a greater proportion of *Bifidobacterium* in the small intestine (*p* < 0.05 for lumen only).

### Isolation of bacterial strains

Lumen and mucosa colon samples from donor 1 produced high numbers of isolates while no colonies were obtained from duodenum and jejunum samples. A total of 40 strains were isolated from the ileum, 125 from ascending colon, 143 from transverse colon and 119 from descending colon (Supplemental Figure S2). Across all intestinal segments, 210 unique strains were isolated from the lumen and 217 from the mucosa. The majority of MRS isolates were classified as *Lactobacillus* (54 from the lumen and 36 from the mucosa) and *Bifidobacterium* (11 from the lumen and 21 from the mucosa). The greatest number of different species, including *Lactobacillus* (53 strains), *Clostridium* (12), *Eggerthella* (12) and *Eubacterium* (12), were isolated from Thioglycolate medium. Finally, of 143 isolates we obtained from Rich media, 103 were *Lactobacillus* and 16 were unclassified.

### Genome sequencing and characterization of L. rhamnosus strains

Eleven *L. rhamnosus* strains were isolated from donor 1 for genome sequencing and further characterization. Representatives from the mucosa of ascending colon, both the lumen and mucosa of ileum, and transverse and descending colon were chosen to determine if the intestinal location impacted strain features (Supplemental Table S5). The average genome size of the strains was 3.05 Mb across an average of 79 contigs. No plasmids, toxin, virulence or antimicrobial genes were detected, and there were no differences between the strains in antibiotic susceptibility tests (Supplemental Table S7). We did not observe specific strain characteristics associated with the location from which they were isolated, instead the strains clustered in two distinct groups composed of two (cluster I) and nine (cluster II) strains. Both strains in cluster I were isolated from the colonic mucosa, whereas the strains in cluster II were isolated from lumen and mucosa and from different intestinal segments. SEM analysis showed that cluster I isolates consistently presented with apparent protuberances in the cell wall (Figure 5C), which may be due to accumulation of material within the cell caused by an inefficiency to transport or phosphorylate sugars (Morabbi Heravi et al., 2019). Isolates from cluster II displayed the classic *Lactobacillus* rod morphology.

Features common to all strains included a putative bacteriocin cluster that contained three core peptides homologous to enterocin X, carnocin CP52 and LSEI_2386, respectively. No modification enzymes were identified; however, six closely located open reading frames (ORFs) associated with bacteriocin immunity and another similar to the transport gene LanT were identified in each cluster. All genomes, with the exception of AMC0710, had at least one CRISPR region comprised of two direct repeats and one spacer region. Corresponding cas genes were not identified (Supplemental Table S8).

RAST (Overbeek et al., 2014) was used to assign genes to specific metabolic subsystems, within which approximately 25 per cent were categorized as “carbohydrate.” Notably, the largest subcategory in all 11 strains was for di- and oligo-saccharide metabolism, including lactose and galactose uptake and utilization, and β-glucoside metabolism. Other enzymes involved in the metabolism of mannose, xylose, fructose, galacturonate, d-glucuronate, deoxyribose, deoxynucleoside and glycogen were also identified (Supplemental Table S9). There were no detectable differences in the profiles of 48 fermentable carbohydrates (Supplemental Table S10).

**Figure 4:**
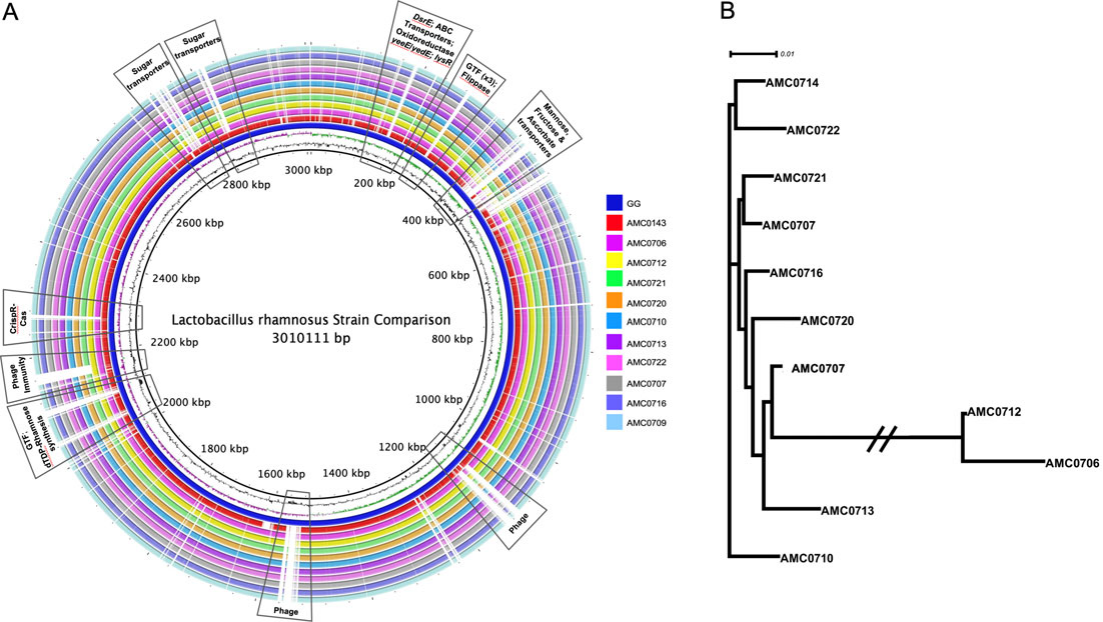
(A) Comparative genomics of the *Lactobacillus rhamnosus* strains aligned against the reference genome, *L. rhamnosus* GG (blue inner ring). Boxes highlight the differences between the clusters and GG. (B) A phylogenetic tree was constructed based on differences in single nucleotide polymorphisms (SNPs) between whole genomes of the strains. cluster I (AMC0712 and AMC0706) is more distantly related to the other isolates (cluster II). The phylogenetic scale is set to 0.01.

A genome alignment using *L. rhamnosus* GG as the reference genome showed that the main differences between the clusters and GG were regarding phage content and the absence in the newly isolated strains of transporters for mannose, fructose and ascorbate (Figure 4A). Also notably absent was the *rfb* operon, whose genes encode for the synthesis of dTDP-rhamnose, which was present in GG but not in the new strains. Conversely, genes present in the two clusters, but not in GG, included different phosphotransferase system (PTS) transporters for mannose, sorbose, fructose, galactitol, galactose, lactose and ascorbate (Supplemental Table S11). Also present in the novel strains but not in GG were β-glucosidase/β-glucoside and ribose transporters, a two-component system, a malolactic regulator and a number of glycosyltransferases and α-galactosidases.

### Comparative genomics analysis of clusters I and II

**Figure 5:**
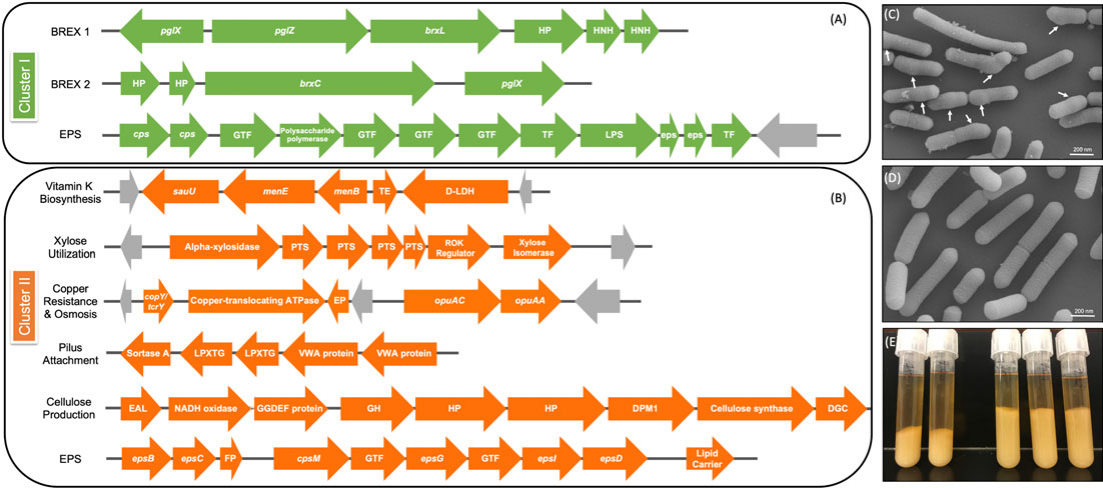
Genomic differences between clusters I and II. (A) Green depicts operons present only in cluster I and (B) orange shows operons present only in cluster II. Grey arrows indicate mobile genetic elements. DGC, diguanylate cyclase; EP, efflux pump; FP, flippase; GH, glycosyl hydrolase; GTF, glycosltransferase; HNH, homing endonuclease; HP, hypothetical protein; TE, thioesterase; TF, transferase. See Supplemental Table S16 for further detail. (C) Scanning electron microscopy (SEM) of *Lactobacillus rhamnosus* AMC0706 and (D) SEM of *L. rhamnosus* AMC0721, representing clusters I and II, respectively. Arrows indicate protuberances in the cell wall of AMC0706. (E) Difference in production of exopolysaccharide (EPS) when cultured in broth between the two clusters.

SNP (Treangen et al., 2014) and ANI analyses revealed that the novel strains clustered into two distinct lineages, designated clusters I and II (Figure 4B). As stated above, the clusters did not reflect the location from which the strains were isolated and were all closely related, with only 0.4 per cent (approximately 148 kbp) difference between them. The genomes in cluster II (3.07 Mb average; *n* = 9) were larger than those in cluster I (2.92 Mb average; *n* = 2). Analysis of coding sequences revealed novel genes in clusters I and II with a greater number of genes present exclusively in cluster II (Figure 5A,B).

The presence or absence of phage components and mobile elements (integrases, recombinases and transposons) accounted for the greatest genomic differences between clusters (Supplemental Tables S12–S14). The two strains in cluster I contained the temperature-sensitive, double-stranded *Lactobacillus casei* phage J1 (Murata, 1971), whereas five strains of cluster II (AMC0721, AMC0722, AMC0710, AMC0707 and AMC0714) carried Lrm1, a 40-kbp temperate phage isolated from an industrial *L. rhamnosus* strain (Durmaz et al., 2008). Both phages belong to the family *Siphoviridae*. There were no cluster-specific differences with respect to CRISPR genes, although cluster I contained two operons encoding genes for the bacteriophage-exclusion BREX system (Hui et al., 2019) (Figure 5A). Phage induction with mitomycin C showed that a previously characterized strain, AMC0143 (Arnold et al., 2017; 2018), which harbors an intact *Staphylococcus* JS01 phage and genes for the *Lactobacillus* phage PLE3, displayed the most notable interruption to growth 5 h post-addition of mitomycin C, confirming optimal inducer concentration. Of the intestinal isolates, only AMC0710, AMC0709 and AMC0720 displayed minor growth interruptions suggesting induction of phage of marginal lysis capacity (Supplemental Figure S3).

We identified a number of putative operons encoding important biological functions present in cluster II, but not in cluster I. These included operons involved in the biosynthesis of vitamin K, utilization of xylose, and a sortase-related cluster of surface proteins involved in pilus-mediated attachment genes (Spirig et al., 2011) (Figure 5B). The cluster II strains also encoded *tcrY*, a gene involved in transferable copper resistance originally identified in *Enterococcus* but common in *Lactobacilli* (Hasman, 2005). Strains AMC0713, AMC0709 and AMC0721 (also in cluster II) may have additional resistance to heavy metals given the presence of two genes for cadmium efflux and a cadmium-transporting ATPase (Supplemental Table S13). At similar locations in the genome scaffolds we identified alternative exopolysaccharide (EPS) operons in each of the clusters. Both contained *epsB* and *epsC* genes at the N-terminus of their respective EPS operons (which was similar but not identical across the clusters), but then diverged with respect to the presence of flippases, glycosyltransfrases and other EPS biosynthesis features. Cluster II also contained an additional operon encoding diguanylate genes involved in biofilm formation and a cellulose synthase, a possible explanation for the increased EPS phenotype observed when cluster II strains were cultured in broth (Figure 5E). Other notable genes found in cluster II but not cluster I included multiple α-galactosidases, a β-glycosyl hydrolase, acetyltransferase, flavodoxin and nitro-reductases, a sodium-dependent transporter and a thymidylate synthase (Supplemental Table S13).

Finally, we performed a comparison analysis of novel regions within clusters to determine differences between strains (Supplemental Figure S4). Differences between strains in cluster II included genes similar to the 2 μm *Saccharomyces* plasmid (Chan et al., 2013), including FLP recombinase, *rep1*, *rep2* and the *rep*-antagonizing factor (RAF) in strains AMC0713, AMC0707 and AMC0720 (Supplemental Table S15). Genes similar to those found in other bacteria included a *mobA*/*mobL* family protein (AMC0713, AMC0721 and AMC0709), a DEAD/DEAH box helicase (all except AMC0713), a thymidylate synthase (AMC0716 and AMC0707) and a cadmium-translocating P-type ATPase (AMC0713, AMC0721 and AMC0709). Between the two strains of cluster I there were minimal differences. AMC0712 contained an extra copy of the gene encoding the cell division protein FtsK, and two hypothetical proteins, whereas AMC0706 had an additonal α-galactosidase, a 5S rRNA and two hypothetical proteins not present in AMC0712.

## Discussion

Using culture-independent and culture-dependent approaches, we determined the microbial profile from lumen and mucosa of intestinal segments from the small intestine and colon of four organ donors. We isolated a total of 427 unique bacterial strains, of which, eleven *L. rhamnosus* strains were selected for further characterization to assess whether the origin or niche within the intestine determined specific genomic or phenotypic characteristics.

Sequenced samples clustered by donor rather than by intestinal segments or regions. This is consistent with previous studies that observed greater variability between individual subjects than sites from which samples were obtained (Stearns et al., 2011), and may be particularly relevant here given the age-range of the donors, a parameter which is known to affect compositon (Xu et al., 2019). Results suggested greater diversity in the small intestine contrary to previous findings (Donaldson et al., 2016; Kastl et al., 2020). However, our data were clearly limited by the number of individuals and samples as well as biased by the high diversity in the small intestine of donors 1 and 4.

The most abundant phylum across all samples and intestinal regions was Firmicutes, consistent with previous observations of populations consuming a Western-style diet, in contrast to individuals consuming a fiber-dense diet, whose microbiomes are composed of a majority of Bacteroidetes (Simpson and Campbell, 2015). The abundance of the genus *Bacteroides* across all samples was also consistent with a gut enterotype common to Western microbiomes (Gorvitovskaia et al., 2016). In contrast, *Prevotella*, a genus common to non-Western microbiomes, was detected in only two samples from the small intestine. Often considered an indicator of microbial dysbiosis (Shin et al., 2015), Proteobacteria were prominent in the intestinal samples of donor 3 suggesting either an unknown pathology or a bloom in Proteobacteria during sample transport and processing.

We observed limited compositional differences between mucosal and luminal samples. As a niche, the mucosal microbiome is poorly characterized. Moreover, shedding of mucus into the lumen can create difficulty in the delineation of luminal from mucosal populations. The healthy mucosa has a higher concentration of oxygen which allows for the radial growth of oxygen tolerant species of Proteobacteria and Actinobacteria (Albenberg et al., 2014). Certain species are also known to utilize the heavily fucosylated glycoproteins of the gut mucus layer, including *Akkermansia muciniphila* (Muriel Derrien, 2004), which are believed to be modulators of human health (Sharon et al., 2018). In our study, *Akkermansia* was poorly represented across all samples, when it has previously been shown to comprise up to 5 per cent of the colonic microbiota (Sharon et al., 2018).

*Bifidobacterium* was detected at a higher abundance in the mucosa compared to the lumen samples, and also in the small intestine compared to colon. This suggests the adaptation of *Bifidobacterium* genus for mucosal adhesion, although a correlation between location and potential beneficial effects remains unknown (He et al., 2001; O’Callaghan and van Sinderen, 2016). In contrast with *Bifidobacterium*, *Lactobacillus* was in general present at greater abundance in the colon compared to the small intestine. In the literature, *Lactobacillus* is often hypothesized to be present in greater abundance in the small intestine, but this has largely been unsubstantiated (Walter, 2008).

It is now recognized that most species of the human gut microbiome can be cultured (Browne et al., 2016), with one study reporting to have isolated 95 per cent of the OTUs detected in 16S rRNA sequencing data, including taxa from the Human Microbiome Project’s “Most Wanted” list (Lau et al., 2016). We identified a total of 12 distinct taxa across three types of media, each previously reported in the human intestine. Despite culturing the samples under strict anaerobic conditions, the lack of obligate anaerobes suggests the exposure of tissues to air may have been enough to suppress oxygen-sensitive members of the gut microbiota (Rolfe et al., 1978).

*L. rhamnosus* has one of the largest pan-genomes amongst the lactic acid bacteria, with an extended variable genome that can enrich a strain with a large repertoire of functions (Ceapa et al., 2016). This genome plasticity is facilitated by a particularly high rate of HGT within this species. Genome analysis of *L. rhamnosus* strains in this study suggests that bacteriophages are one of the major forces driving the transfer of genetic material, and probably extending the genetic capability of the strains, since phage elements, transferable genes (transposons, mobile elements and integrases) and phage defense mechanisms accounted for the largest proportion of genomic differences. In fact, phage content was one of the key differences between clusters. Cluster I contained the virulent J1 phage, five strains of cluster II harbored Lrm1, which has previously been proposed to be a defective phage due to incomplete lysis of cultures *in vitro* but was hypothesized to provide immunity to superinfection from subsequent phage attack (Durmaz et al., 2008).

One of the key phenotypic differences between the two clusters was the increased volume of EPS produced by cluster II when cultured in liquid medium. EPS has been shown to provide protection for luminal strains and facilitate biofilm formation, while also aiding in cell adhesion (Deo et al., 2019). Composition of the EPS operon differed between the two strain clusters, and cluster II contained an additional operon for cellulose biosynthesis, which is likely to contribute to the extracellular matrix. Cluster II also contained a DEAD box helicase, which has been shown to be important in cell aggregation in *Lactobacillus reuteri* (Roos et al., 1999). Furthermore, a class C sortase operon, functioning as a pilin polymerase (Spirig et al., 2011), was only present in cluster II, offering further evidence that the strains in this cluster may have an advantage for intestinal adhesion.

Another attribute present in cluster II, which may be beneficial for the host, was the putative ability to biosynthesize vitamin K. Production of this metabolite has been observed in lactic acid and other gut bacteria (Morishita et al., 1999; Ramotar et al., 1984). Although not definitively proven, the production of bacterially derived Vitamin K, ie. menaquinone, may be of health benefit to the host in lieu of dietary-sourced phylloquinone (Beulens et al., 2013).

A number of differences were also observed within each cluster. The most relevant are several genes in cluster II, which appear to have originated from a 2 μm *Saccharomyces* plasmid, an efficient self-replicator which has no known effect on the phenotype of its natural host and has been likened to an intracellular parasite (Futcher et al., 1988; Reynolds et al., 1987). In *Saccharomyces*, several of these genes work together to control plasmid replication; *FLP* is responsible for the amplification of the plasmid, while *RAF* promotes expression of *FLP*. Both genes are negatively regulated by the plasmid partitioning proteins *rep1*/*rep2* (Murray et al., 1987). Although it would seem unlikely that *Lactobacillus* possesses the scaffolding or infrastructure to stably incorporate or express such genes, their presence is nonetheless interesting when considering a potential interkingdom gene transfer. Although most studies of such transfer events have focussed on the uptake of genetic content by eukaryotes from prokaryotes, demonstrating that conjugation is possible between bacteria and yeast (Stachel and Zambryski, 1989), genetic transfer in the opposite direction is not as well-established. *Lactobacillus* and *Saccharomyces*, however, have a demonstrated close relationship in nature (Marsh et al., 2013; Megee et al., 1972).

Although a great deal has been learned in recent years by using feces as a proxy for GI tract communities, there remains much to learn about bacterial distribution within the intestinal tract. Results presented here substantiate previous studies showing not just inter-individual variability but also variation in genera along the intestine. Furthermore, despite its limitations regarding the number of subjects and strains selected for further characterization, our study suggests that strains with specific adaptations were not niche-restricted, but instead had extended plasticity and differed in biologically relevant genetic traits, including adhesion potential, environmental resistance and metabolite production.

## Research transparency and reproducibility

The 16S rRNA amplicon reads for individual donors are available under biosample accession numbers SAMN16111581- SAMN16111584. *Lactobacillus* genomes have been deposited to Genbank/NCBI under BioProject PRJNA645702, and are publicly available in the Sequence Read Archive (SRA) under accession numbers SRX9105356–SRX9105366. The manuscript is our own original work, and does not duplicate any other previously published work. The manuscript has been submitted only to the journal and it is not under consideration, accepted for publication or in press elsewhere. Manuscripts were not deposited on pre-print servers; all listed authors know of and agree to the manuscript being submitted to the journal; and the manuscript contains nothing that is abusive, defamatory, fraudulent, illegal, libellous, or obscene.

## Supplementary material

For supplementary material accompanying this paper visit https://doi.org/10.1017/gmb.2020.4. click here to view supplementary material
