# Supplementary material for: Lumen and mucosa-associated Lactobacillus rhamnosus from the intestinal tract of organ donors
Source: Gut Microbiome (Camb). 2020 Nov 10;1:e4. doi: 10.1017/gmb.2020.4 (PMC11406413; doi:10.1017/gmb.2020.4)
Supplement: Supplementary file 1 [file gmbsup.zip › S2632289720000043sup001.pptx]

## Slide 1
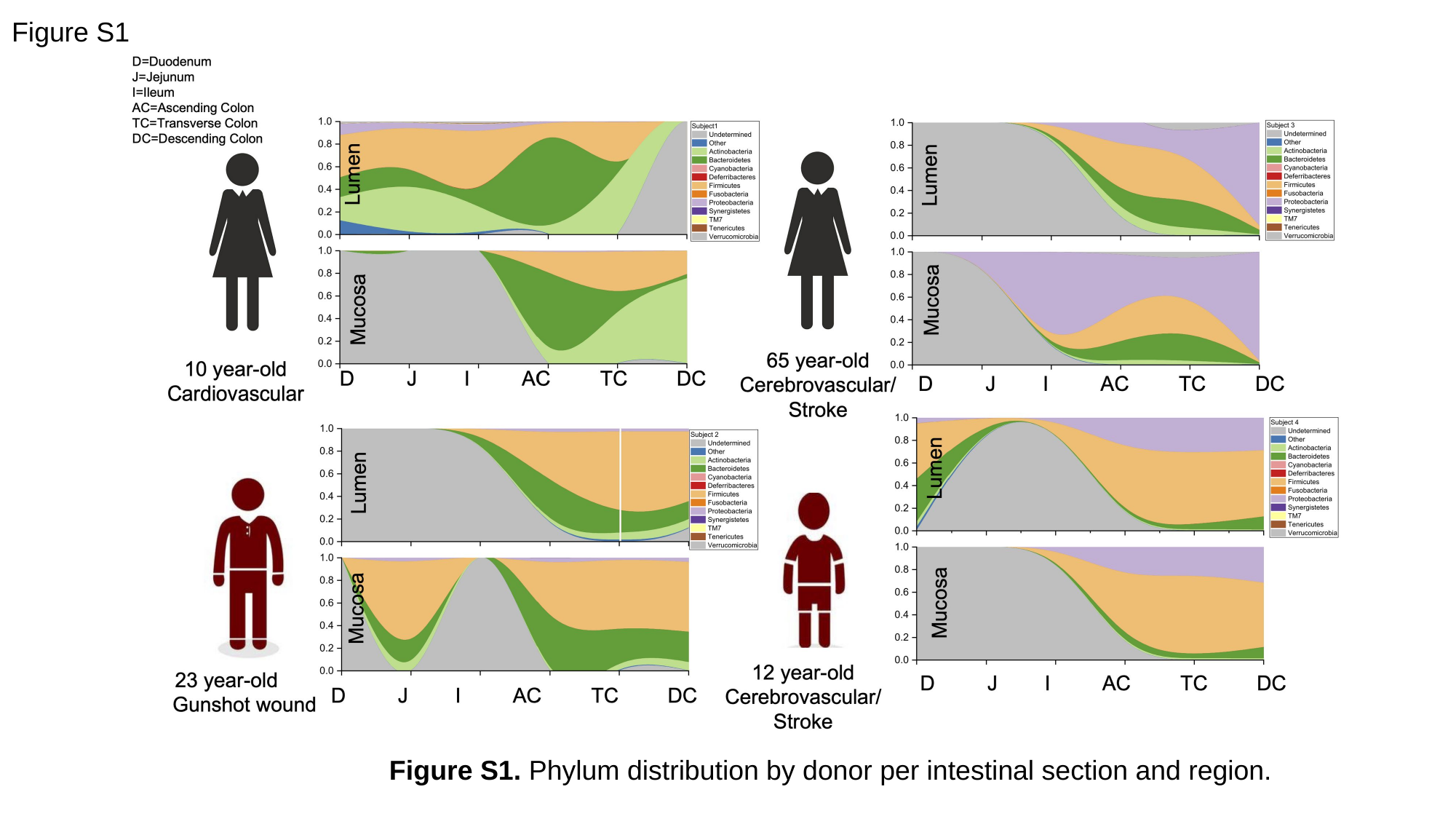

Figure S1
Figure S1. Phylum distribution by donor per intestinal section and region.

## Slide 2
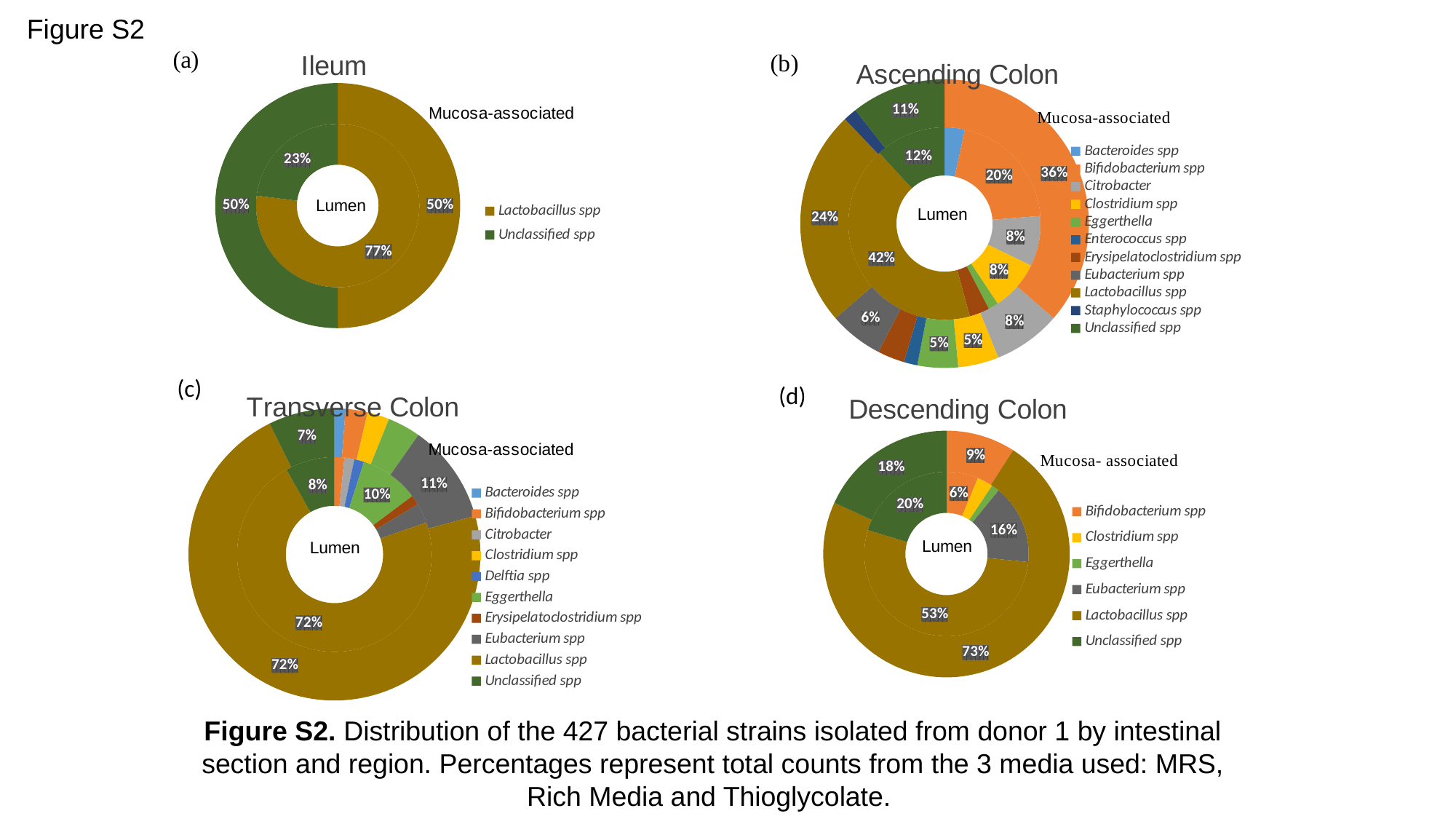

### Chart: Ileum
| Category | Ileum  | Ileum  |
|---|---|---|
| Bacteroides spp | None | None |
| Bifidobacterium spp | None | None |
| Citrobacter | None | None |
| Clostridium spp | None | None |
| Delftia spp | None | None |
| Eggerthella | None | None |
| Enterococcus spp | None | None |
| Erysipelatoclostridium spp | None | None |
| Eubacterium spp | None | None |
| Lactobacillus spp | 20.0 | 7.0 |
| Staphylococcus spp | None | None |
| Unclassified spp | 6.0 | 7.0 |Figure S2
### Chart: Ascending Colon
| Category | Ascending Colon | Ascending Colon |
|---|---|---|
| Bacteroides spp | 2.0 | None |
| Bifidobacterium spp | 12.0 | 24.0 |
| Citrobacter | 5.0 | 5.0 |
| Clostridium spp | 5.0 | 3.0 |
| Delftia spp | None | None |
| Eggerthella | 1.0 | 3.0 |
| Enterococcus spp | None | 1.0 |
| Erysipelatoclostridium spp | 2.0 | 2.0 |
| Eubacterium spp | None | 4.0 |
| Lactobacillus spp | 25.0 | 16.0 |
| Staphylococcus spp | None | 1.0 |
| Unclassified spp | 7.0 | 7.0 |
### Chart: Transverse Colon
| Category | Transverse colon | Transverse colon |
|---|---|---|
| Bacteroides spp | None | 1.0 |
| Bifidobacterium spp | 1.0 | 2.0 |
| Citrobacter | 1.0 | None |
| Clostridium spp | None | 2.0 |
| Delftia spp | 1.0 | None |
| Eggerthella | 6.0 | 3.0 |
| Enterococcus spp | None | None |
| Erysipelatoclostridium spp | 1.0 | None |
| Eubacterium spp | 2.0 | 9.0 |
| Lactobacillus spp | 44.0 | 59.0 |
| Staphylococcus spp | None | None |
| Unclassified spp | 5.0 | 6.0 |
### Chart: Descending Colon
| Category | Descending Colon | Descending Colon |
|---|---|---|
| Bacteroides spp | None | None |
| Bifidobacterium spp | 4.0 | 5.0 |
| Citrobacter | None | None |
| Clostridium spp | 2.0 | None |
| Delftia spp | None | None |
| Eggerthella | 1.0 | None |
| Enterococcus spp | None | None |
| Erysipelatoclostridium spp | None | None |
| Eubacterium spp | 10.0 | None |
| Lactobacillus spp | 34.0 | 40.0 |
| Staphylococcus spp | None | None |
| Unclassified spp | 13.0 | 10.0 |(c)
(d)
Figure S2. Distribution of the 427 bacterial strains isolated from donor 1 by intestinal section and region. Percentages represent total counts from the 3 media used: MRS, Rich Media and Thioglycolate.

## Slide 3
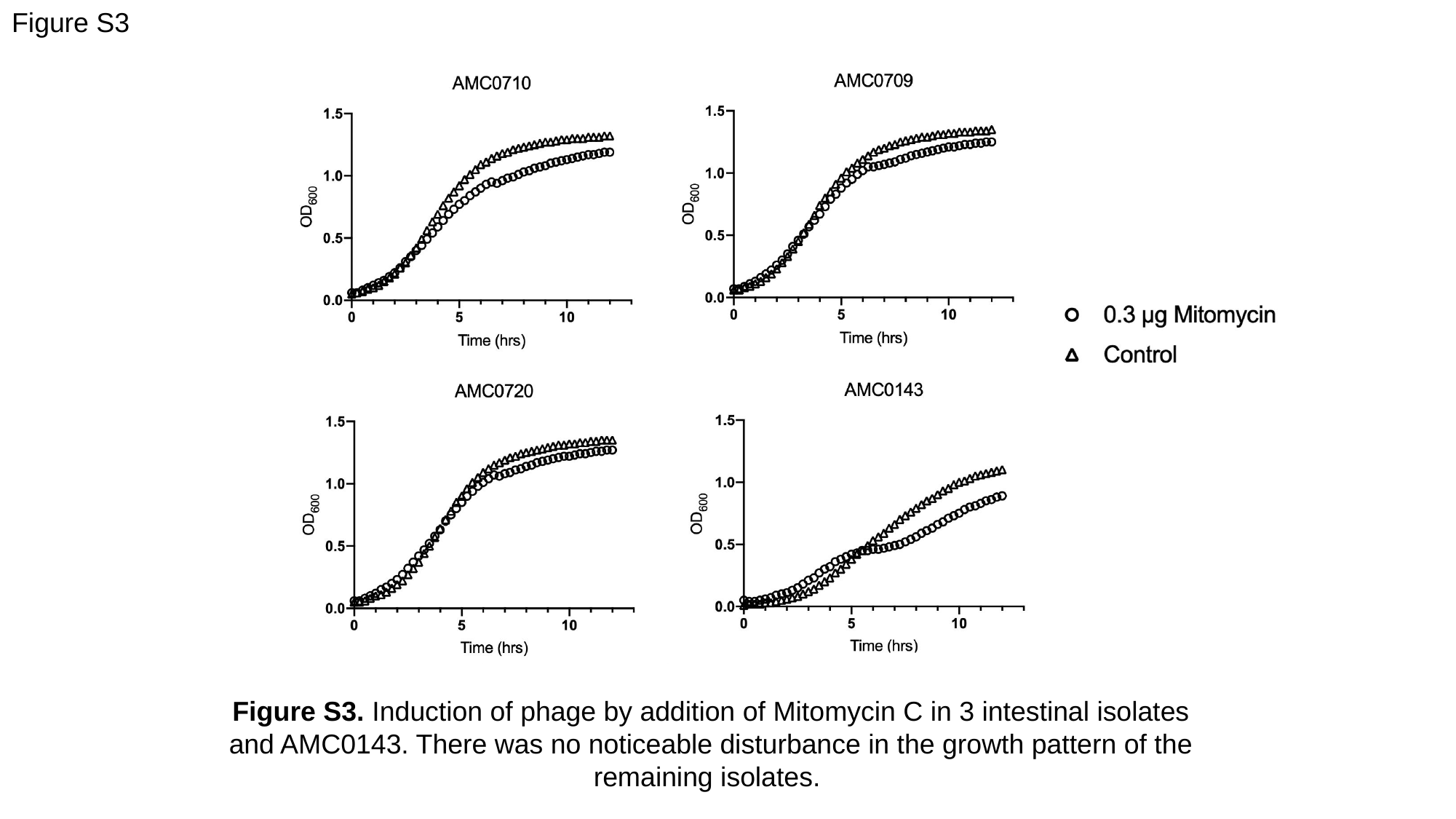

Figure S3
Figure S3. Induction of phage by addition of Mitomycin C in 3 intestinal isolates and AMC0143. There was no noticeable disturbance in the growth pattern of the remaining isolates.

## Slide 4
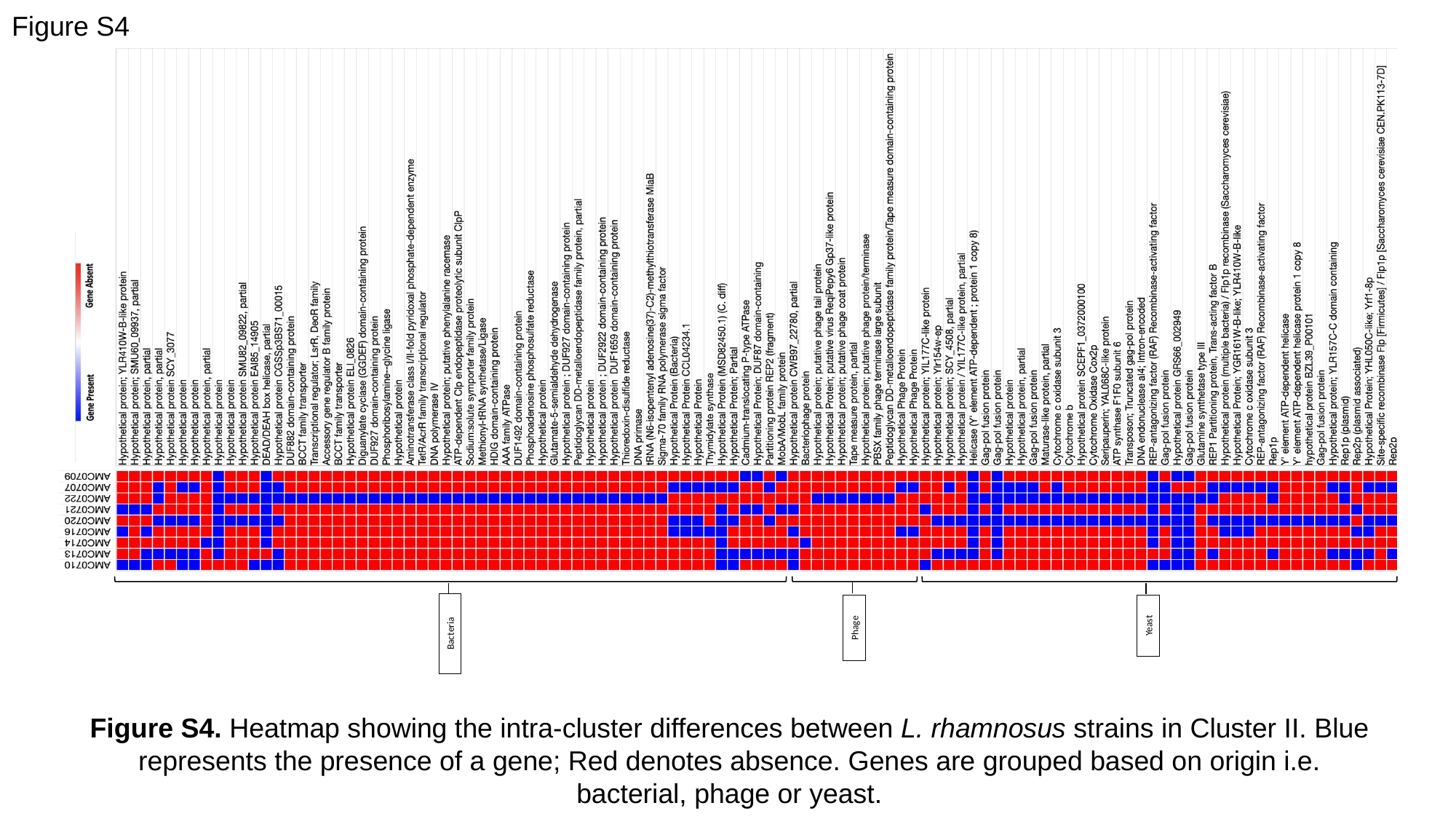

Bacteria
Phage
Yeast
Figure S4
Figure S4. Heatmap showing the intra-cluster differences between L. rhamnosus strains in Cluster II. Blue represents the presence of a gene; Red denotes absence. Genes are grouped based on origin i.e. bacterial, phage or yeast.
