# Supplementary figures and images for: Lumen and mucosa-associated Lactobacillus rhamnosus from the intestinal tract of organ donors
Source: Gut Microbiome (Camb). 2020 Nov 10;1:e4. doi: 10.1017/gmb.2020.4 (PMC11406413; doi:10.1017/gmb.2020.4)

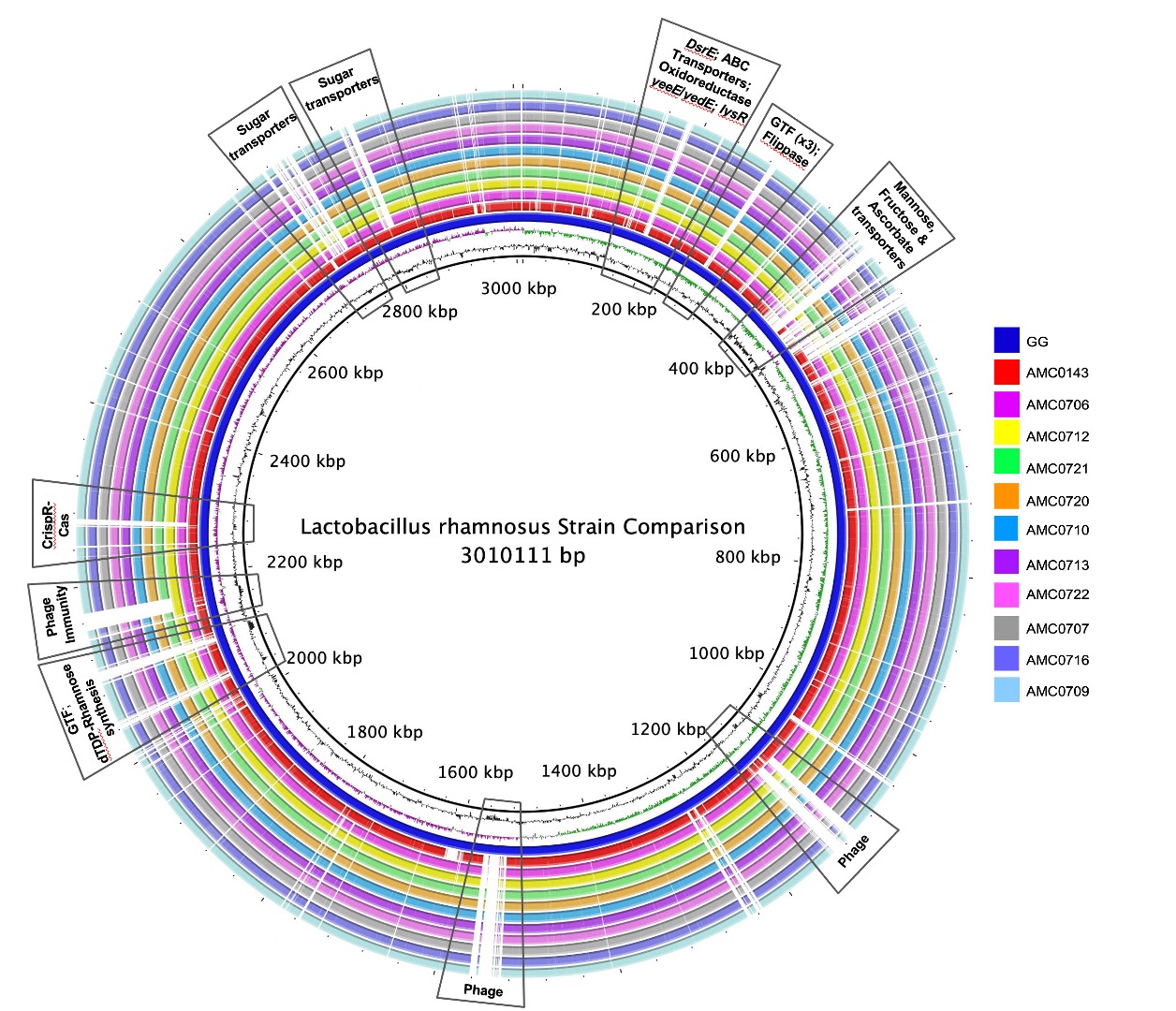

Supplement: Supplementary file 1 [file gmbsup.zip › S2632289720000043sup003.jpg]
